# Modified Natural Rubber as a Simple Chemical Sensor with Smartphone Detection for Formaldehyde Content in a Seafood Sample

**DOI:** 10.3390/molecules27072159

**Published:** 2022-03-27

**Authors:** Chonnipa Yeerum, Piyanat Issarangkura Na Ayutthaya, Kullapon Kesonkan, Kanokwan Kiwfo, Ploenpit Boochathum, Kate Grudpan, Monnapat Vongboot

**Affiliations:** 1 Department of Chemistry, Faculty of Science, King Mongkut’s University of Technology Thonburi, Bangkok 10140, Thailand; chonnipa.yeerum@gmail.com (C.Y.); piyanat.tp@gmail.com (P.I.N.A.); kullapon.kesonkan@gmail.com (K.K.); iplothum@hotmail.com (P.B.); 2 Center of Excellence for Innovation in Analytical Science and Technology for Biodiversity-Based Economic and Society (I-ANALY-S-T_B.BES-CMU) and Department of Chemistry, Faculty of Sciences, Chiang Mai University, Chiang Mai 50200, Thailand; k.kanokwan11@gmail.com

**Keywords:** modified natural rubber, biodegradable platform, chemical sensor, smartphone, formaldehyde, seafood

## Abstract

A new biodegradable platform-based sensor for formaldehyde assay is proposed. Natural rubber latex was modified to polylactic acid–chloroacetated natural rubber polymer blend sheets. The polymer blend sheet was grafted using a water-based system with amine monomers as a platform, with a spot exhibiting positive polarity for immobilizing with anionic dye (Acid Red 27). The sensor was exposed to formaldehyde. The color intensity of the dye on the sensor spot would decrease. Using a smartphone with image processing (via ImageJ program), the color intensity change (∆B) could be followed. A linear calibration, ∆B intensity = 0.365 [FA] + 6.988, R^2^ = 0.997, was obtained for 10–150 mM FA with LOD and LOQ at 3 and 10 mM, respectively (linear regression method). The precision was lower than 20% RSD. Application to real seafood samples was demonstrated. The ready-to-use sensor with the proposed method was cost-effective, was portable for on-site analysis, and demonstrated green chemical analysis.

## 1. Introduction

Formaldehyde (FA) has been associated with aspects of health for human beings. FA is referred to as a carcinogen by the International Agency for Research on Cancer of the World Health Organization [1]. FA may appear in food by natural occurrence or by being added as a food preservative to increase the shelf life of some products, including fruits, dried food, fish, and seafood [2,3,4].

In a related report, recommendations for daily intake of FA were not more than 0.2 or 0.15 mg kg^−1^ of body weight, according to the United States Environmental Protection Agency and WHO, respectively, while the Italian Health Department in 1985 proposed FA limitations in cod and shellfish aquatic products as 60 mg kg^−1^ and 10 mg kg^−1^, respectively [3].

Various techniques have been employed to quantify FA. High-performance liquid chromatography (HPLC) and gas chromatography–mass spectrometry (GC-MS) were reported to determine FA. HPLC with pre-column derivatization with 2,4-dinitrophenylhydrazine (DNPH) enables the determination of FA with sensitivity benefits. The procedures were applied to tap water, rainwater, beer, fruit juice, and squid samples [5,6,7,8]. Samples were pre-treated before being introduced to HPLC with UV detection. GC-MS was employed with similar sample pre-treatment and with DNPH pre-column derivatization to determine FA in squid and squid products [9]. FA adulteration in seafood has garnered increasing interest. For less complicated procedures and instrumentation, spectrometric techniques have been developed since the 1950s. They are based on Nash reagent, involving the reactions of acetylacetone, ammonia, and FA to form 3,5-diacetyl-1,4-dihydrotoludine, which is associated with fluorescence and colorimetry [10].

Colorimetric procedures employing Nash as a color reagent for FA have been developed for fish samples [11,12]. Recently, a digital camera has been used as an alternative detector instead of a conventional colorimeter to determine FA using Nash reagent for seafood samples with natural-material-based platforms [13,14]. Nash also serves as a fluorescence reagent for FA with the use of paper-based platforms [15,16]. Chromotropic acid and disodium salt dihydrate have been used as color reagents for FA [17,18,19].

Recently, a paper-based platform for FA assay was proposed for food samples [20]. It has been adapted from conventional titration, whereby FA could be quantified by treating with excess sodium sulfite to produce sodium hydroxide, being further titrated with a standardized sulfuric acid solution for the end point of color change (blue to colorless) of thymolphthalein as an acid-base indicator [21,22].

Primary amine-containing thin films, obtained by copolymerization of a primary amine monomer (N-(3-aminopropyl)methacrylamide hydrochloride) with co-monomers, N,N′-methylene-bis-acrylamide, acrylamide, and 2,2′-azobisisobutyronitrile (AIBN)) on a pattern-printed microscope slide, with sorption of an anionic dye (Acid Blue 92, tartrazine, Acid Red 112, indigo carmine, chrysophenine, and Acid Red 27), were reported. The microscope slide with the dye spot, after exposure to FA, was measured for absorbance change using a spectrophotometer. The absorbance change related to the FA concentration [23].

Recently developed chemical analysis techniques for food and food safety analysis include downscaling platforms such as strip arrays, microplates, and microfluidic devices with various modes of detection, or even simple sensors. Such development offers advantages including on-site analysis, especially with the possibility of using a smartphone for detection [24,25].

As natural rubber is hugely produced in this region, with its natural biopolymer properties, this work endeavored to develop greener chemical analysis by introducing a biodegradable platform of a modified natural rubber to replace the microscope slide glass-based material. The polymerization of amine monomers and crosslinkers of natural rubber sheets were produced using a simpler water-based system in normal atmosphere conditions before immobilizing an anionic dye to obtain a ready-to-use sensor. Moreover, using a smartphone for detection instead of a spectrophotometer would offer several advantages. The biodegradable platform would serve as a simple ready-to-use chemical sensor, enabling convenient, rapid, and on-site assay of FA in a seafood sample.

## 2. Results and Discussion

### 2.1. The Sensor

**Figure 1:**
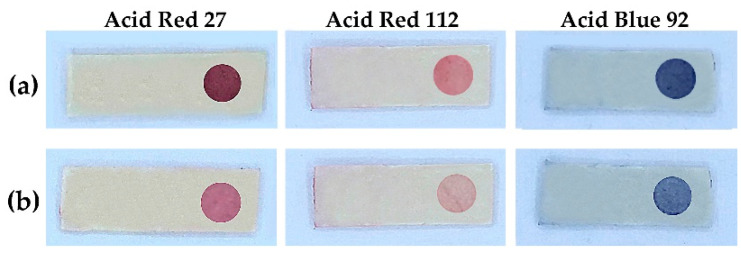
Sensor platform: (**a**) immobilized with anionic dyes; and (**b**) after treatment with 100 mM FA. (Sensor platform size: 0.1 × 0.5 × 2.0 cm).

The new sensor for the FA assay was obtained from modifying natural rubber latex (NRL) for a polymer base of polylactic acid–chloroacetated natural rubber polymer blend grafted with two monomers providing a solid biodegradable platform, with amine functional group linked to an anionic dye to serve as a sensor spot on the polymer-based platform (see Figure 1). When FA interacts at the sensor spot, the anionic dye will be released from the sensor spot. Color intensity changes at the sensor spot could be sensed using a smartphone camera. With image processing, the color intensity would relate to the quantity of FA.

The platform of the sensor was fabricated using two main steps, namely, modifying NRL to polylactic acid–chloroacetated natural rubber polymer blend sheets and grafting polymerization. To modify the NRL, the polymerization used formic acid, hydrogen peroxide, and chloroacetic acid to obtain chloroacetated natural rubber (CNR). It was followed by blending with the components of interest, i.e., CNR, polylactic acid (PLA), corn starch, and silica. The polymer blend should result in the properties contributed from the blending components, whereby CNR contributes elasticity, with PLA and silica contributing strength, leading a thin sheet being formed, while corn starch and silica would contribute to the polarity property and whiter sheet background for color contrast when observing the color change. In addition, the biodegradable solid platform should enable green chemical analysis.

The ratios of the components, in the form CNR: PLA: corn starch: silica, varied by weight: (1) 30:70:30:0, (2) 40:60:40:0, (3) 40:60:30:0, (4) 40:60:40:3, and (5) 40:60:30:3, respectively. Ratio #(4) was chosen as providing reasonable desired properties of the platform, including 1 mm thickness and homogeneous white sheets. The obtained platform was appropriate when immobilizing with a dye (Acid Red 27) (see Figure S1).

To introduce the amine functional group in the polylactic acid–chloroacetated natural rubber polymer blend platform, the obtained polymer blend was grafted with two amine monomers, namely, APMA and AAm. It was expected that AAm would provide the amorphous property of the grafted polymer, while APMA would provide the amine functional group, offering a link to an anionic dye. This plays important roles in the interaction with FA. For a polylactic acid–chloroacetated natural rubber polymer blend platform of 0.5 cm × 2 cm, a spot with surface area of 0.3 cm diameter was grafted by irradiating with UV-C under normal atmosphere. The UV irradiation would activate the double-bonds of the polymer blend for crosslink. The polylactic acid–chloroacetated natural rubber blend served as crosslinker and initiator by itself. With our design, the irradiation box could accommodate 100 pieces for each irradiation and with varied irradiation times (2, 4, 6, 8, and 10 h). For 8 h or longer irradiation, platform cracking was observed, while for 4 h or less irradiation, the target spot was observed to be wet. Consequently, irradiation for 6 h was chosen.

Comparing IR spectra of unmodified and modified surface spots (see Figure S2), the peaks due to the amine group (N–H bending at 1652–1537 cm^−1^ and ${\mathrm{NH}}_{2}$ and N–H wagging vibrations at 1218 cm^−1^) were pronounced in the modified surface spot.

Mechanisms involving the above have been of interest and should be further investigated.

In the present work, only two instead of four monomers were used in the preparation of the primary amine-containing thin films on a pattern-printed microscope slide in a related report [23]. The two common monomers in both works were APMA and AAm, but the initiator (2,2′-azobisisobutyronitrile (AIBN)) and crosslinker (N,N′-methylene-bis-acrylamide) were not used in this work. Furthermore, in the related study, the grafting was performed using DMSO solvent and under nitrogen atmosphere, while this work used an underwater-based system and normal atmosphere. This reduced the use of toxic chemicals and led to greener chemistry. In addition, this new biodegradable platform-based sensor for FA offered an advantage due to the waste produced by the previously reported sensor using a microscope glass slide.

The polylactic acid–chloroacetated natural rubber polymer blend platform could easily be immobilized by soaking the platform in an aqueous solution of anionic dye. The active area (3 mm diameter) with grafted polymer spot posting amine functional group would link to the anionic dye, which would be released when interacting with FA. This could be due to the imine formation from Schiff base reaction [26]. The nucleophiles due to the amine group (${\mathrm{NH}}_{2}$) introduced in the polymer blend based at the surface spot sensor would interact with the induced positive charge (*δ*+) from carbon atoms of carbonyl groups of FA to form the imine compounds (C=N compounds) (as illustrated in Figure S3).

**Figure 2:**
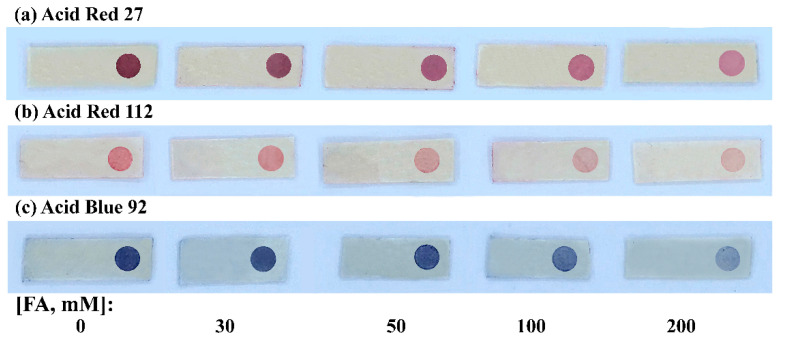
Sensor platforms immobilized with: (**a**) Acid Red 27, (**b**) Acid Red 112, and (**c**) Acid Blue 92, treated with FA.

In this work, three anionic dyes of red and blue, namely, Acid Red 27, Acid Red 112, and Acid Blue 92, were piloted, as the color intensity change should be more sensitive compared with a yellow anionic dye when applying a smartphone camera for detection. Red, Green, Blue (RGB) color mode was applied for image processing. As expected, for sensors using the red dyes (Acid Red 27 and Acid Red 112), B intensity was found to be suitable, while R intensity would be appropriate for sensors using the blue dye (Acid Blue 92). From Figure 2, even with the naked eye, the color changes at the sensor spots could be observed more sharply for Acid Red 27 than the others. From the color intensity profiles (Figure S4), leading to calibration graphs (Figure S5), the calibration with B intensity values for Acid Red 27 provided the highest slope with reasonable R^2^ value. Regarding calibration use for the FA assay, ∆B intensity obtained by the B intensity was due to the FA concentration subtracted by the B intensity due to blank color. Following that, the FA sensor comprised the polylactic acid–chloroacetated natural rubber polymer blend platform, with the sensor spot of grafted polymer hosting the amine functional group linking the Acid Red 27 dye.

**Figure 3:**
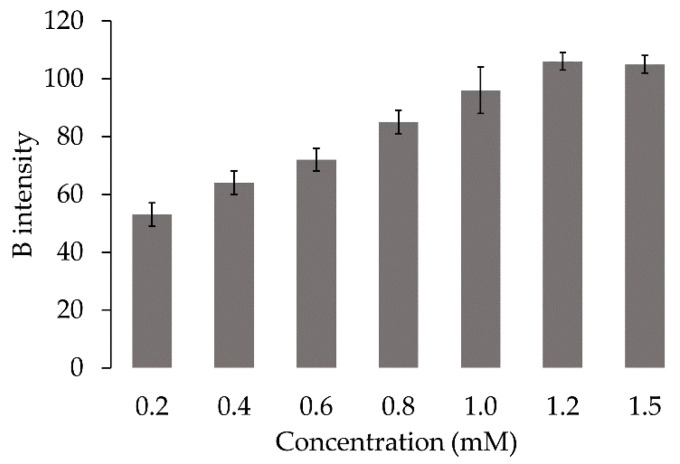
Effect of Acid Red 27 concentration.

Immobilizing Acid Red 27 solutions (0.2–1.5 mM) were studied for the FA sensor. According to the results (Figure 3), the dye solution of 1.2 mM or more provided constant B intensity. The FA sensor could be obtained by soaking in a 1.2 mM Acid Red 27 solution for 30 min. Each of the sensors was checked for quality control by considering whether the B intensity of the immobilized Acid Red 27 sensor spot had a value within 10% RSD of the mean, allowing it to serve as a ready-to-use sensor for the FA assay.

### 2.2. Sensing and Evaluation for FA

The colorimetric assay of FA involves pH of analyte solution and exposure condition.

#### 2.2.1. Effect of pH

To investigate the effect of pH that would play a role in the interaction due to ${\mathrm{NH}}_{3}^{+}$, the amine group (${\mathrm{NH}}_{2}$) and the dye, 50 mM FA solutions with pH of 3, 7, 8, 9, and 10 (adjusted by 0.1 M HCl and/or 0.2 M NaOH) were studied by 650 rpm stirring for 30 min. An increase was observed in ΔB intensity with the pH value until 8, after which it decreased. A borate buffer (boric acid/borate) of pH 8 was used for further experiments.

#### 2.2.2. Exposing Condition

The effects of contact time and stirring speed were studied using the ready-to-use sensor for FA (10, 50, and 100 mM), under the previous conditions (pH 8 with borate buffer). Contacting times of 1, 3, and 5 min were studied, with a constant stirring speed of 650 rpm. Good linearities were observed for 3 and 5 min contacting times, but not for 1 min, as lower ΔB intensity resulted from the higher concentration of FA. Exposure time of 3 min was selected for further work. Investigation of stirring speed was conducted by varying between 500, 650, and 800 rpm, under the previous conditions, with 3 min exposing time. All the stirring speeds resulted in the same ΔB intensity.

#### 2.2.3. Analytical Performance

##### Statistical Treatment in Connecting to the Evaluation

The statistical treatment for evaluation in using the ready-to-use sensor pieces was investigated by considering the concept previously reported [27]. The sensor pieces were tested for 10–150 mM FA by applying the conditions obtained above. For one FA concentration, five sensor pieces were applied. Five values of ∆B intensities were obtained from the five sensor pieces. The ∆B intensity values were treated for statistical values (Q-test, mean, percentile, and median), and the results are summarized in Table S1. For each set of five ∆B intensity values, the median was evaluated. The medians of ∆B intensities were plotted against concentrations (10, 50, 100, and 150 mM FA) for a calibration graph. Statistical treatment applying percentile ranks (20th–80th) resulted in the same value as the median. When treating the five values of the ∆B intensities, the obtained mean after outlier treatment using the Q-test was found to be the same value as the median value. Using the median values of ∆B intensities instead of the mean values for plotting a calibration graph is a less complicated procedure, resulting in faster evaluation.

By doing that, the procedure offers five replications for one sample analysis.

##### Analytical Characteristics

A calibration graph was obtained by plotting of median of ∆B intensity against FA concentration. A linear calibration was obtained in the range of 10–150 mM FA, ∆B intensity = 0.365 [FA] + 6.988, R^2^ = 0.997. The LOD and LOQ were 3 and 10 mM, respectively, calculated using the linear regression method [28]. The relative standard deviation (RSD) was less than 20%.

##### Interferences Study

Studies were made for interferences by the two groups: the species with carbonyl group having potential due to imine formation and anion species co-existing in a sample. Acetone and acetaldehyde were selected for the former, while chloride and phosphate for the latter. A concentration of FA of 70 mM, which is in the middle of the calibration graph, was mixed with the potential interfering species. If the ∆B intensity due to the mixture of 70 mM FA with the potential interfering species fell within ±10% RSD of that of the FA alone, the potential interfering species would not be considered to interfere. From the study, it was found that the maximum concentrations of 0.1 M acetone, 0.1 M acetaldehyde, and 1 M chloride did not interfere, while 7 g L^−1^ for phosphate did.

### 2.3. Applications to Real Sample

The developed method was demonstrated to determine FA in real samples. The samples were fresh squid (SQ), a concentrate solution (CS), and a dilute solution for the preservation of seafood on a market shelf (MS). The results obtained by the proposed procedure were compared with those of the reference titration method. The results are summarized in Table 1. By applying the t-test, the result of each sample obtained by the proposed method did not significantly differ with that of the reference method at 95% confidence level (t_calculated_ less than t_critical_ (1.943), df *=* 6). The recovery study was made by spiking FA standards in the samples. The recoveries were 96–120% and 92–103% for CS1 and CS2, respectively.

Notably, the developed sensor was aimed at screening FA content in a sample using simple procedures, including the sample preparation. The sensitivity could not be comparable to those methods with high sensitivity, but the developed method should be useful for onsite analysis at marketplace. Using a smartphone should offer additional advantages in traceability of the on-site analysis.

## 3. Materials and Methods

### 3.1. Chemicals and Reagents

All chemicals were of analytical grade, unless otherwise stated. Deionization (DI) water was used throughout the experiment.

Low-ammonia natural rubber latex (60% of dry rubber content) was obtained from Num Rubber & Latex Co., Ltd., Bangkok, Thailand.

The other chemicals included formic acid (85% *w/w*, Fisher Chemical, Leicestershire, UK), hydrogen peroxide (35% *w/w*, Ajax Finechem, New South Wales, Australia), chloroacetic acid (99% *w/w*, Sigma-Aldrich, St. Louis, MO, USA), polylactic acid (PLA) (grade 4043D, NatureWorks Co., Ltd., Minneapolis, MN, USA), silica (VN-3, 15 µm, United Silica (Siam) Co., Ltd., Rayong, Thailand), corn starch from Ocean Foods Co., Ltd., Bangkok, Thailand, N-(3-aminopropyl)methacrylamide hydrochloride (APMA) (Sigma-Aldrich, Darmstadt, Germany), acrylamide (AAm) (Vivantis, Selangor Darul Ehsan, Malaysia), and anionic-azo dyes (Acid Red 112, Acid Red 27, and Acid Blue 92) (TCI, Tokyo, Japan).

A stock solution of 10 M formaldehyde (FA) was prepared from FA solution (37% *w/w*, Ajax Finechem, New South Wales, Australia) and was stored in a refrigerator. Working standard solutions of FA were prepared daily by appropriate dilution of the stock solution with DI water.

A 0.1 M borate buffer solution at pH 8 was prepared from boric acid ($H_{3}{\mathrm{BO}}_{3}$, Merck, Darmstadt, Germany) and sodium borate decahydrate (${\mathrm{Na}}_{2}B_{4}O_{7}$ ° 10$H_{7}O$, Ajax Finechem, New South Wales, Australia).

### 3.2. Fabrication of Sensor

**Figure 4:**
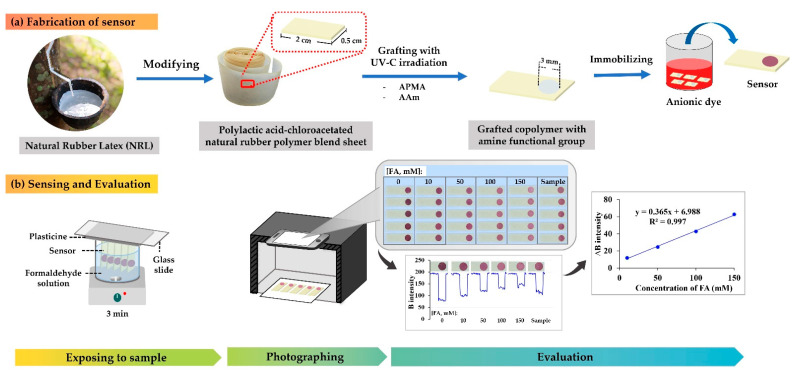
Illustration of the developed sensor for FA assay: (**a**) fabrication and (**b**) sensing and evaluation.

Fabrication of the sensor involved three steps (see Figure 4a), namely: 1. modifying NRL to polylactic acid–chloroacetated natural rubber polymer blend sheet; 2. grafting the polymer blend sheet with 2 amine monomers; and 3. immobilizing anionic dye onto sensor spot of the modified surface of the polymer sheet platform.

Polylactic acid–chloroacetated natural rubber polymer blend sheet was obtained by mixing NRL, hydrogen peroxide, formic acid, chloroacetic acid, corn starch, polylactic acid, and silica in the appropriate ratio by adjusting the ratio from related work [29]. Using an internal mixer (MX500-D75L90-TQ, Chareon Tut Co., Ltd., Samutprakarn, Thailand) and using a two-roll mill (Chaicharoen Karnchang Co., Ltd., Bangkok, Thailand), 1 mm thickness, 20 cm width, and 3 m length of polylactic acid–chloroacetated natural rubber polymer blend sheets was produced, which then was cut into small pieces (0.5 × 2 cm).

After that, the polymer blend sheet was grafted by dropping 15 µL of an aqueous mixture of APMA (30 mM) and AAm (5 mM) (resulting in the ratio of 0.45:0.075 µmol, respectively) onto each polylactic acid–chloroacetated natural rubber polymer blend piece. The pieces were then irradiated for 6 h with 18 watts UV-C lamp (Thai Inter Lamp Co., Ltd., Samutprakarn, Thailand) in a lab-made UV-C lightbox (25 × 57 × 69 cm) with a switchboard (KJL, Thailand). The obtained polymer-based platform was rinsed with DI water. The dried polymer-based platform was soaked in an anionic dye solution for 30 min. The platform was then rinsed with DI water. The dye-immobilized pieces should serve as a ready-to-use sensor for FA assay.

### 3.3. The Procedure for FA Assay Using the Proposed Sensor with a Smartphone and Image Processing

Figure 4b illustrates the procedure for quantification of FA. A set of five sensor pieces was used for one exposure. An arrangement for the sensor piece exposing the analyte solution was made by having one end of the piece attached to a glass plate using plasticine, while immersing the other end with the sensor spot in the analyte solution (standard/sample) and stirring at 650 rpm for 3 min. The exposed sensors were allowed to dry at room temperature. Then, digital images of the sensor pieces were taken using a smartphone (iPhone 6S, Apple Inc., Cupertino, CA, USA) and the Yamera application (AppMadang, Version 3.9) on manual mode: ISO 100, Zoom 3x, and Focus 0.6, under the light-controlled box (UDIOBOX UDIO BIZ 40 × 40 × 40 cm)**.** The images were processed for the RGB color system using ImageJ Software (National Institutes of Health, Bethesda, MD, USA). RGB intensity profiles were obtained. The results obtained from five sensor pieces were statistically considered (as described in Section 2.2.3. Analytical Performance; Statistical Treatment in Connecting to the Evaluation) for plot calibration graph.

### 3.4. Sample Preparation

Sample preparation was adapted from related reports [20,30,31]. A liquid sample was filtered with Whatman No. 1 filter paper to remove particles. For the fresh squid sample, a weighed portion was blended for 1 min with water (200 mL). The mixture was centrifuged at 6000 rpm for 10 min. An aliquot (50 mL) of the supernatant was added with 95% *v*/*v* ethanol (5 mL) and stirred for 5 min to precipitate proteins. The resulting mixture was again centrifuged for another 10 min at 6000 rpm. A clear supernatant could be observed. An aliquot (20 mL) of the supernatant was taken and treated with borate buffer to adjust pH and volume to be 8 and 25 mL, respectively, and was ready to be used for the FA assay.

### 3.5. Reference Method

The reference titration method was followed with modification from [20]. Briefly, 10 mL of the treated sample was pipetted and mixed with 10 mL of 1 M sodium sulfite solution in an Erlenmeyer flask. Then, three drops of phenolphthalein as the indicator were added. The titrant was titrated by standardized sulfuric acid with end point of color change from pink to colorless.

## 4. Conclusions

A new biodegradable sensor for FA assay was developed. Natural rubber latex was modified with hydrogen peroxide, formic acid, chloroacetic acid, corn starch, polylactic acid, and silica to polylactic acid–chloroacetated natural rubber polymer blend to serve as a solid platform. The polymer blend was grafted under simpler condition by UV-C irradiation at normal atmosphere with an aqueous mixture of two primary monomers (APMA and AAm). The positive polarity surface at the sensor spot from amine functional group would link with the anionic dye (Acid Red 27), which would be released when interacting with FA. Color intensity changes at the sensor spot could be sensed using a smartphone camera. Via image processing, the color intensity would relate to the quantity of FA. With the statistic treatment of the color intensity signals, a median value was meaningful for a calibration graph plot (a plot of median color intensity value versus FA concentration), and application to seafood samples was demonstrated. The developed ready-to-use sensor offered advantages in being cost-effective, portable, and traceable for on-site analysis. This enables green chemical analysis.

## Figures and Tables

**Table 1:** Assays of FA contents in samples.

Samples ^1^	Concentration of FA (mM)		t_calculated_
	Proposed Method (*n* = 5)	Reference Method [20] (*n* = 3)	
SQ1	32 ± 7	31 ± 1	0.24
SQ2	26 ± 4	29 ± 1	1.24
SQ3	36 ± 7	37 ± 1	0.24
CS1	52 ± 6	56 ± 1	1.11
CS2	70 ± 6	75 ± 1	1.39
MS	ND	ND	−

^1^ SQ: squid sample; CS: concentrate solution for preservation of seafood on a market shelf; MS: solution for the preservation of seafood on a market shelf. ND = Not detectable.

## Data Availability

All the data are reported in this manuscript and Supplementary Materials.

## Supplementary Materials

The following supporting information can be downloaded at: https://www.mdpi.com/article/10.3390/molecules27072159/s1, Figure S1: Intensities of photos taken from the immobilized polylactic acid–chloroacetated natural rubber polymer blend with the ratios of CNR: PLA: corn starch: silica being: (1) 30:70:30:0; (2) 40:60:40:0; (3) 40:60:30:0; (4) 40:60:40:3, and (5) 40:60:30:3 by weight (Experimental condition of the grafting polymerization with irradiation time for 4 h and immobilized with 0.8 mM Acid Red 27); Figure S2: IR spectra of the polylactic acid–chloroacetated natural rubber polymer blend platforms (CNR: PLA: corn starch: silica: 40:60:40:3 by weight): (a) unmodified surface; (b) modified surface with amine functional group; Figure S3: Illustration of the sensing reaction; Figure S4: R, G, B intensity profiles: (a) Acid Red 27, (b) Acid Red 112, and (c) Acid Blue 92 obtained from Figure 2a–c, respectively; Figure S5: Calibration plots (intensity vs. FA concentration): (a) Acid Red 27, (b) Acid Red 112, and (c) Acid Blue 92 obtained from Figure S4a–c, respectively; Table S1: Statistical treatment for evaluation of images of the sensors for FA determination.

## Sample Availability

Samples of the polylactic acid–chloroacetated natural rubber polymer blend grafted with two amine monomers are available on reasonable request from the corresponding authors.
