# Supplementary material for: Modified Natural Rubber as a Simple Chemical Sensor with Smartphone Detection for Formaldehyde Content in a Seafood Sample
Source: Molecules. 2022 Mar 27;27(7):2159. doi: 10.3390/molecules27072159 (PMC9000404; doi:10.3390/molecules27072159)
Supplement: Supplementary file 1 [file molecules-27-02159-s001.zip › molecules-1636949-supplementary.pdf]

molecules

MDPI

Supplementary Materials

Modified Natural Rubber as a Simple Chemical Sensor with Smartphone Detection for Formaldehyde Content in a Seafood Sample

Chonnipa Yeerum [1], Piyanat Issarangkura Na Ayutthaya [1], Kullapon Kesonkan [1], Kanokwan Kiwfo [2], Ploenpit Boochathum [1], Kate Grudpan [2,*] and Monnapat Vongboot [1,*]

[1] Department of Chemistry, Faculty of Science, King Mongkut's University of Technology Thonburi, Bangkok 10140, Thailand; chonnipa.yeerum@gmail.com (C.Y.); piyanat.tp@gmail.com (P.I.N.A.); kullapon.kesonkan@gmail.com (K.K.); iplothum@hotmail.com (P.B.)

[2] Center of Excellence for Innovation in Analytical Science and Technology for Biodiversity-Based Economic and Society (I-ANALY-S-T_B.BES-CMU) and Department of Chemistry, Faculty of Sciences, Chiang Mai University, Chiang Mai 50200, Thailand; k.kanokwan11@gmail.com

* Correspondence: kgrudpan@gmail.com (K.G.); sumalee.tan@kmutt.ac.th (M.V.)

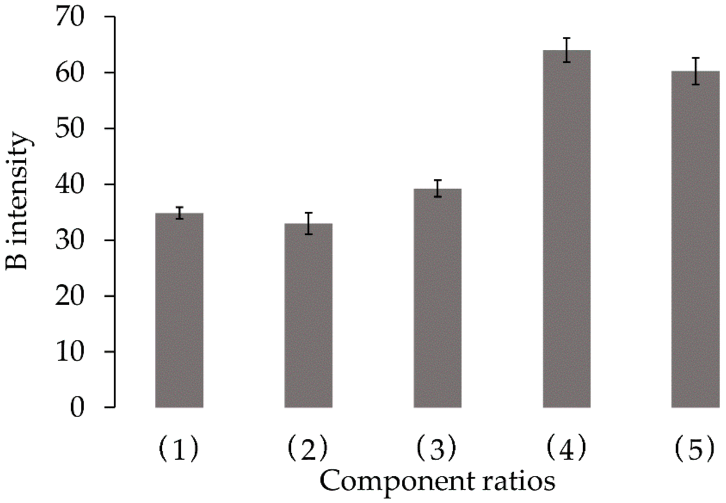

Figure S1. Intensities of photos taken from the immobilized polylactic acid-chloroacetated natural rubber polymer blend with the ratios of CNR: PLA: corn starch: silica being: (**1**) 30:70:30:0; (**2**) 40:60:40:0; (**3**) 40:60:30:0; (**4**) 40:60:40:3 and (**5**) 40:60:30:3 by weight (Experimental condition of the grafting polymerization with irradiation time for 4 h and immobilized with 0.8 mM Acid Red 27).

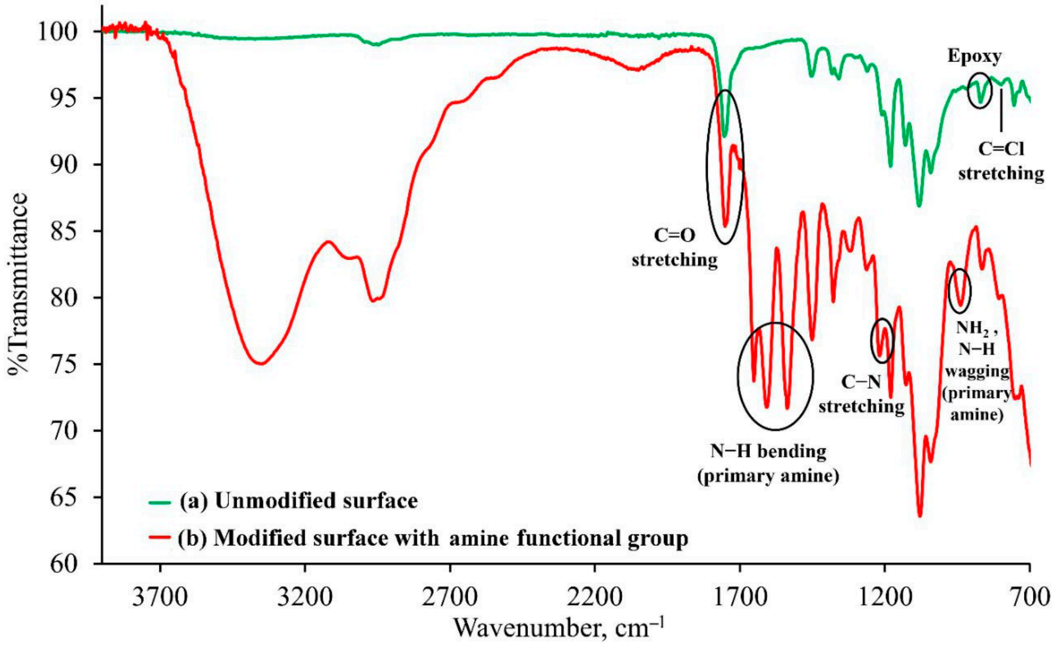

Figure S2. IR spectra of the polylactic acid-chloroacetated natural rubber polymer blend platforms (CNR: PLA: corn starch: silica: 40:60:40:3 by weight): (**a**) unmodified surface; (**b**) modified surface with amine functional group (see text).

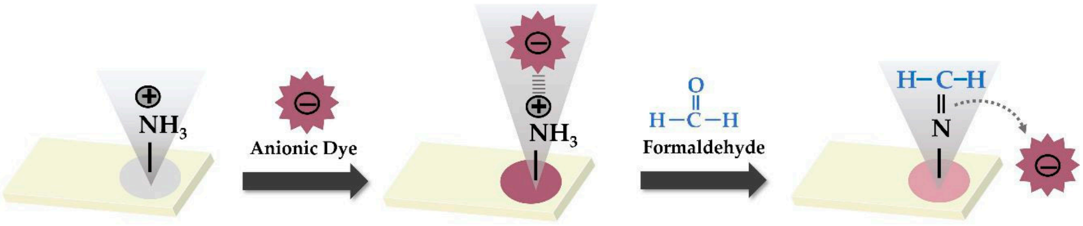

Figure S3. Illustration of the sensing reaction.

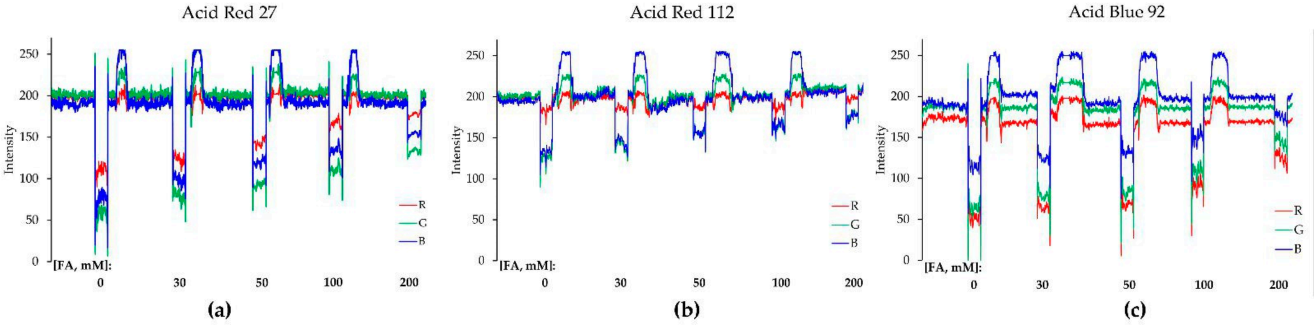

Figure S4. R, G, B intensity profiles: (**a**) Acid Red 27, (**b**) Acid Red 112, and (**c**) Acid Blue 92 obtained from Figure 2a–c, respectively.

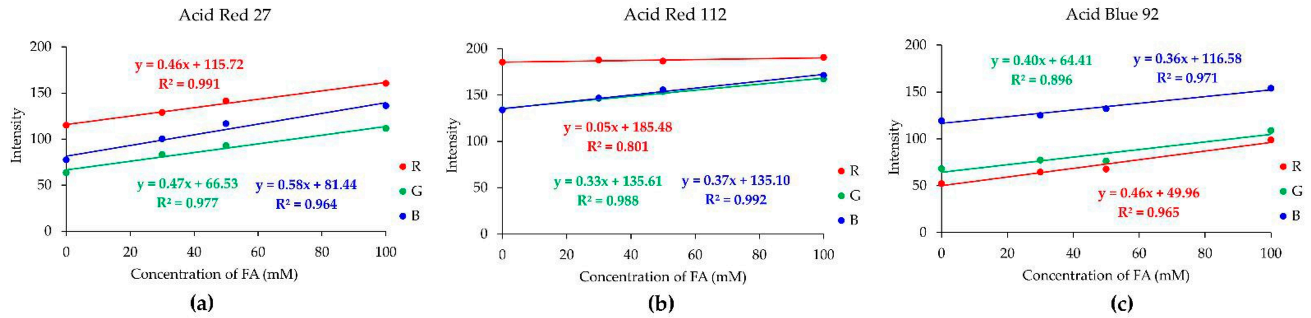

Figure S5. Calibration plots (intensity vs FA concentration): (**a**) Acid Red 27, (**b**) Acid Red 112, and (**c**) Acid Blue 92 obtained from Figure S4a–c, respectively.

Table S1. Statistical treatment for evaluation of images of the sensors for FA determination.

Concentration of FA (mM)	ΔB Intensity	Ranking of ΔB Intensity	Mean without Q-test	Median Value	Percentile Rank	After Outlier (P_{20}–P_{80})	Mean (P_{20}–P_{80})	Mean After Q-test
	8.4	7.5			17			
	11.8	8.4			33	8.4		
10	7.5	11.8	12	12	50	11.8	11	12
	13.1	13.1			67	13.1		
	16.7	16.7			83			
	25.5	9.8			17			
	9.8	22.0			33	22.0		
50	24.0	24.0	21	24	50	24.0	24	24
	22.0	24.5			67	24.5		
	24.5	25.5			83			
	45.8	40.4			17			
	43.5	42.7			33	42.7		
100	40.4	42.7	43	43	50	42.7	43	43
	42.7	43.5			67	43.5		
	42.7	45.8			83			
	64.0	54.1			17			
	62.6	61.9			33	61.9		
150	63.6	62.6	61	63	50	62.6	63	63
	54.1	63.6			67	63.6		
	61.9	64.0			83			
